# Metacestodosis as a threat for endangered species: a fatal case of neurocoenurosis in an Apennine chamois (*Rupicapra pyrenaica ornata* Neumann, 1899) housed in an *ex-situ* conservation facility within the Monti Sibillini National Park (Italy)

**DOI:** 10.1016/j.ijppaw.2026.101251

**Published:** 2026-06-16

**Authors:** Benedetto Morandi, Stefano Gavaudan, Silvia Crotti, Deborah Cruciani, Giacomo Rossi, Alessio Angorini, Fabrizio Dini, Alessandro Rossetti, Federico Morandi

**Affiliations:** a Istituto Zooprofilattico Sperimentale dell’Umbria e delle Marche “Togo Rosati”, via G.Salvemini, 1, Perugia, 06126, Italy; b School of Biosciences and Veterinary Medicine, University of Camerino, Via Circonvallazione 93/95, Matelica, MC, 62024, Italy; c Monti Sibillini National Park, P.zza del Forno 1, Visso, 62039, Italy

**Keywords:** Caprinae, Wildlife, Threatened species, Parasites, Tapeworms, Taeniids, *Taenia multiceps*

## Abstract

The complex life cycles of taeniid parasites rely strictly on predator-prey interactions across multiple hosts. Among these, *Taenia multiceps* causes neurocoenurosis, a neglected but often fatal neurological disease with a worldwide distribution. While well-documented in livestock, its impact on vulnerable, fragmented wildlife populations remains poorly understood, despite carrying a significant zoonotic risk to humans. This study describes a fatal case of neurocoenurosis in an endangered Apennine chamois (*Rupicapra pyrenaica ornata*) housed in an *ex-situ* conservation facility within the Monti Sibillini National Park (Central Italy). The diagnosis was established through a comprehensive diagnostic workflow combining live clinical evaluation, post-mortem histopathology of intracranial cysts, and molecular confirmation via PCR. Pathological and molecular testing confirmed the presence of *T. multiceps* coenuri within the brain tissue. These findings demonstrate that this neglected parasite can act as a direct mortality factor for the IUCN Red Listed Apennine chamois. Given the increasing spatial overlap between traditional pastoral systems and wild ungulates, wildlife surveillance is critical to monitor this threat and prevent impactful demographic consequences on endangered subspecies.

## Highlights

• First description of *Taenia multiceps* infection in the Apennine chamois.
• High-pressure parasitic infections represent a substantial threat to endangered species.
• Environmental overlap can facilitate the spillover of parasitic infection between domestic and wild species.
• To perform necropsy on wildlife is essential for the local and global understanding of epidemiology of generalist parasites.

## Graphical abstract

**Figure:**
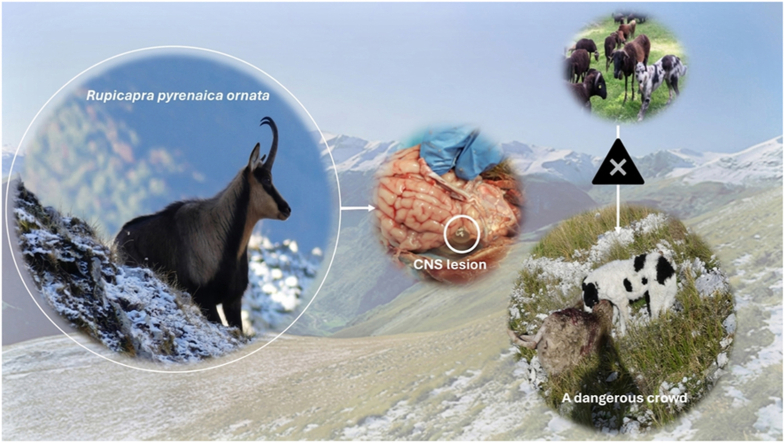

## 1 Introduction

*Taenia multiceps*, the causative agent of neurocoenurosis, follows a predator-prey life cycle involving domestic and wild canids as definitive hosts and ungulates, mainly sheep and goats, as intermediate hosts (Varcasia et al., 2022). Its epidemiology is commonly related to pastoral context where traditional sheep farming persists (Vasileiou et al., 2015). Canids acquire the infection by consuming brain or spinal cord tissues containing larval cysts from infected carcasses, whereas small ruminants contract the disease by ingesting tapeworm eggs shed in canine feces. (Chubb et al., 2020). In such a scenario, shepherds play a crucial role in the spread of neurocoenurosis, as the practice of leaving infected sheep central nervous system (CNS) tissues in the environment after on-farm slaughter - where dogs can freely access and consume them - promotes the persistence and dissemination of the parasite (Morandi et al., 2020). In addition, *T. multiceps* represents a risk in a public health perspective, given its zoonotic role, with a fatality rate of around 42% among human cases (Deplazes et al., 2019).

Interactions between domestic and wild hosts, that share a high degree of environmental overlap, can facilitate the transmission and maintenance of parasitic infections (Bengis et al., 2002), especially in hyperendemic areas. Therefore, the role of wildlife in the epidemiology of *T. multiceps* is receiving growing attention. So, wildlife surveillance is crucial to monitor for human and animal health since global distribution and flexibility in intermediate hosts of many and even more critical taeniids species may enlarge their host range (Morandi et al., 2021). In endangered species, high-pressure parasitic infections represent a substantial threat as they can lead to increased mortality and morbidity, especially in populations characterized by low genetic diversity and small demographic sizes. In such a context, even minor health disturbances may have significant population-level consequences (Pedersen et al., 2007).

The Apennine chamois (*Rupicapra pyrenaica ornata* Neumann, 1899) is an endemic subspecies to the central Apennines and is currently listed as “vulnerable” in the red list of threatened species by the International Union for Conservation of Nature (IUCN, 2018) due to its limited distribution and small and fragmented populations. This subspecies is also strictly protected under Italian and international laws (L.157/92, CITES Appendix II). The most severe population bottleneck occurred in the early 20th century, specifically around the 1940s, when the population dropped to fewer than 30 individuals, all confined to the Abruzzo, Lazio, and Molise National Park (COORNATA Project, 2015). Many reintroduction projects were carried out in several protected areas in Central Italy, according to the Italian Action Plan for the Apennine chamois (Duprè et al., 2001). In 2008, 13 Apennine chamois were reintroduced into the Monti Sibillini National Park (MSNP). Between 2010 and 2014, with further support from the European project LIFE Coornata ('Development of coordinated protection measures for Apennine chamois'—LIFE09 NAT/IT/000183), an additional 18 individuals were released (Rossetti et al., 2015). Since that time, owing to sustained conservation efforts, the population has exhibited a continuous upward trend (Bocci et al., 2016) until November 2025 counting approximately 500 individuals (Rossetti, pers. comm.), though it remains vulnerable.

This study describes the first documented case of fatal *T*. *multiceps* infection in an Apennine chamois housed in an *ex-situ* conservation facility within the MSNP in the Municipality of Bolognola (Macerata – Marche region), supported by clinical presentation, histopathological findings, and molecular identification of the parasite.

## 2 Material and methods

### 2.1 Clinical evaluation

An 8-month-old Apennine chamois (*Rupicapra pyrenaica ornata*), born in an *ex-situ* conservation facility (fenced area of approximately 4 ha) within the MSNP (42.998870N; 13.222648E), was referred to the Veterinary Teaching Hospital of the University of Camerino in March 2021 with neurological symptoms that had been worsening for approximately 15 days. The initial clinical signs reported by the park rangers included depressed sensorium, incoordination, and decreased appetite. Consequently, the animal was admitted to the hospital's wildlife department and started on a therapeutic regimen consisting of multivitamin and selenium supplements (Selevit® 0.4 ml/kg IM, q24h; Fatro S.p.A., Bologna, Italy) and hypertonic solution boluses (NaCl 7.5%; B. Braun Milano S.p.A., Milan, Italy) every 12 h. Due to the further deterioration of its neurological condition, the animal underwent brain Magnetic Resonance Imaging (MRI).

### 2.2 MRI evaluation

Sedation was achieved via intramuscular administration of xylazine (0.2 mg/kg; Nerfasin®, Dechra S.r.l., Turin, Italy; 100 mg/ml) and ketamine (5 mg/kg; Ketavet®, MSD Animal Health S.r.l., Segrate, Milan, Italy; 100 mg/ml). Fifteen minutes post-administration, an intravenous cannula was placed in the cephalic vein for fluid and drug delivery. General anesthesia was induced and maintained via face mask using isoflurane (IsoFlo, Zoetis S.r.l., Rome, Italy) in pure oxygen (FiO_2_ > 0.8) under spontaneous breathing. MRI of the brain performed with T1, T2, FLAIR, and 3D T1 sequences.

### 2.3 Necropsy and histopathology

A complete necropsy was performed by placing the animal in dorsal recumbency and proceeding according to the standard technique used for small ruminants. Specifically, the central nervous system was examined by opening the skull, revealing the presence of a large cyst located at the level of the central nervous system (CNS). The brain was then extracted, the vertebral canal was opened, and the spinal cord was examined to assess whether other similar lesions were present. Samples were taken from all organs, including the brain and spinal cord. All tissues sampled for histopathological examination were fixed in 10% buffered formalin, then dehydrated in a progressive alcohol series, and embedded in paraffin. Subsequently, the tissues were cut into 3 μm thick sections and placed on electrostatically charged slides (*Histoline*®, Milan, Italy). The sections were then stained with hematoxylin and eosin for basic histo-morphological examination, and specific histochemical stains such as modified Gram for bacteria localization in tissues (Engbaek et al., 1979), PAS staining for glycoproteins, mucosubstances, glycogen/amylopectin, and Ziehl-Neelsen staining for the detection of acid- and alcohol-fast substances and bacteria. The slides were examined under a light microscope (*Olympus*®, Germany) at different magnifications.

### 2.4 Molecular analysis

The Exgene™ FFPE Tissue DNA kit (GeneAll Biotechnology Co.®, Seoul, Korea) was used to extract genomic DNA from the paraffin-embedded cerebral cyst section. The DNA was then amplified by a multiplex-polymerase chain reaction (mPCR), using GoTaq® G2 Flexi DNA Polymerase (Promega, Madison, WI, USA) and primer pairs Cest4/Cest 5, Cest3/Cest5, and Cest1/Cest2 to target *E. granulosus* rrnS gene (117 bp), *Taenia* spp. rrnS gene (267 bp), and *E. multilocularis* nad1 gene (395 bp), respectively (Trachsel et al., 2007). Electrophoresis on 2% agarose gel stained with Midori Green Advance (NIPPON Genetics®, Düren, Germany) was performed to visualize the amplicon obtained through UVITEC Essential V6 system (Thermo Fisher Scientific, Waltham, MA, USA). Subsequently, it was purified by QIAquick PCR Purification Kit (QIAGEN®, Hilden, Germany) and subjected to Sanger sequencing with Cest5_seq_ primer (Trachsel et al., 2007) using BrilliantDye™ Terminator v3.1 Cycle Sequencing Kit (NimaGen®, Nijmegen, The Netherlands). A 3500 Genetic Analyzer (Applied Biosystems®, Foster City, CA, USA) generated the electropherogram. Using the BioEdit Sequence Alignment Editor software version 7.2.5 (accessed on 08 September 2023), the resulting sequence was trimmed at the ends and then aligned in the GenBank database.

## 3 Results

Upon initial clinical examination, the animal exhibited depressed sensorium, tachycardia, and tachypnea. The accessible mucous membranes were pale, and the animal was afebrile. Neurological examination revealed severe incoordination, an ataxic gait, and persistent bilateral horizontal nystagmus (see supplemental material). However, no clinical improvement was observed despite the initial therapeutic regimen. At the MRI evaluation, a cystic lesion was observed showing hyperintense signal on T2-weighted images and hypointense signal on T1-weighted images, with complete suppression on FLAIR sequences. The lesion was located along the third and fourth ventricles, which appeared compressed and dilated. There was ventrodorsal compression of the cerebellum. Findings were suggestive of a cystic lesion of probable parasitic origin (see supplemental material). Due to the progressive deterioration of the clinical condition and poor prognosis, the Apennine chamois was euthanized.

The necropsy examination revealed, the presence of a macroscopic cystic formation, 3.5 cm in diameter, with translucent walls, visible only in the upper half, partially penetrating the brain tissue, with the presence of sterile microabscesses and a limited area of cerebral necrosis. Briefly, upon opening the skull, an area of meningeal thickening and turbidity with a focal distribution was observed. The cyst was located on the roof, between the cerebellum and the left cerebral hemisphere (Fig. 1). Parasitological examination of the cyst revealed whitish spots on the transparent wall of the cyst and on the germinal membrane, representing the scolices. Small foci of purulent exudate were observable adjacent to the cyst, at the level of the leptomeninges; in the same areas, there were also areas of hemorrhage and necrosis of the nervous tissue in close proximity to the cyst. The lungs appeared congested and edematous, with very dark areas, especially on the hypostatic side. Upon section of the parenchyma, copious oozing of fluid mixed with blood was observed from both the parenchyma and the airways. Examination of the other organs appeared mostly normal, with some alterations of a chronic nature-scarring changes in the liver, which showed some scarring and focal fibrosis within the parenchyma, corresponding to the visceral surface of the organ, resulting from previous parasitic migratory lesions. Based on the clinical presentation and autopsy findings, chronic coenurosis was preliminarily suspected. Histopathological evaluation of the tissue sections revealed a picture of multifocal pyogranulomatous meningoencephalitis, accompanied by eosinophilic infiltrations. No bacteria were isolated following bacterial culture of the brain tissue. Besides the cyst, which was found macroscopically, no other small cysts were detected histopathologically within the brain parenchyma or in the spinal cord. Histologically, the cyst revealed a three-layered wall (cuticular, cellular, adventitia), surrounded by chronic inflammation. In particular, outer cuticular layer appeared laminated, acellular, and eosinophilic; inner cellular layer consistent of nucleated cells, and adventitious layer, represented by a fibrous capsule formed by fibroblasts and collagen of the host reaction (Fig. 2A and D). An intense inflammatory reaction was observed, focally, around the cyst, associated with congestion, hyperemia, edema, and abundant infiltration of mononuclear cells, characterized by lymphocytes but also numerous plasma cells with a greatly expanded, PAS+, Golgi apparatus. These cells were accompanied by the presence of eosinophilic granulocytes and multinucleated giant cells, mostly of the foreign body type, surrounding the cyst wall. The granulomatous lesions were located near the cyst, with necrotic debris at the center, surrounded by epithelioid macrophages and giant cells, both Langhans- and foreign body-type (Fig. 2B, C, 2E). In some cases, amorphous eosinophilic material, Splendore-Hoeppli-type, was found, forming radiating clusters (Fig. 2F). Perivascular cuffing and gliosis were generally observed in the CNS; more rarely, small areas of compressive-origin of spongiosis, neuronal vacuolation, neuronal necrosis, and neuronophagia were observed around the multifocal granulomatous foci. Some areas showed infarction with prominent necrosis and formation of granulomas, representing probable aborted parasitic lesions, showing central necrotic-amorphous material of parasitic origin (Fig. 2F).

**Fig. 1:**
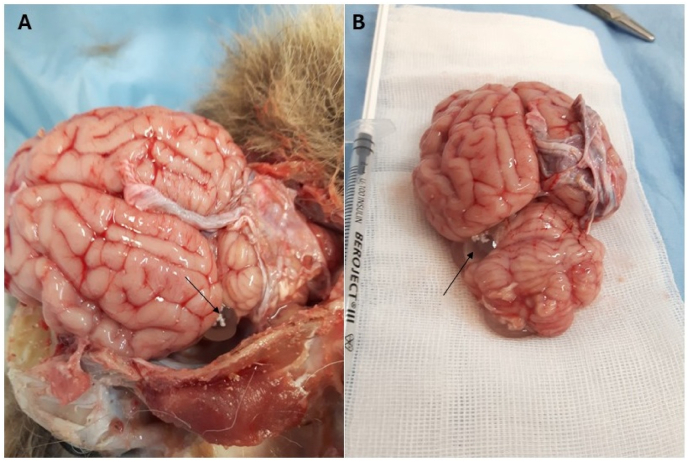
Macroscopical evidence of the fluid-filled bladder cyst (black arrows) (3.5 cm in diameter) detected between the cerebellum and the left cerebral hemisphere during the necropsy in the Apennine chamois; (A) left-lateral view; (B) caudal view.

**Fig. 2:**
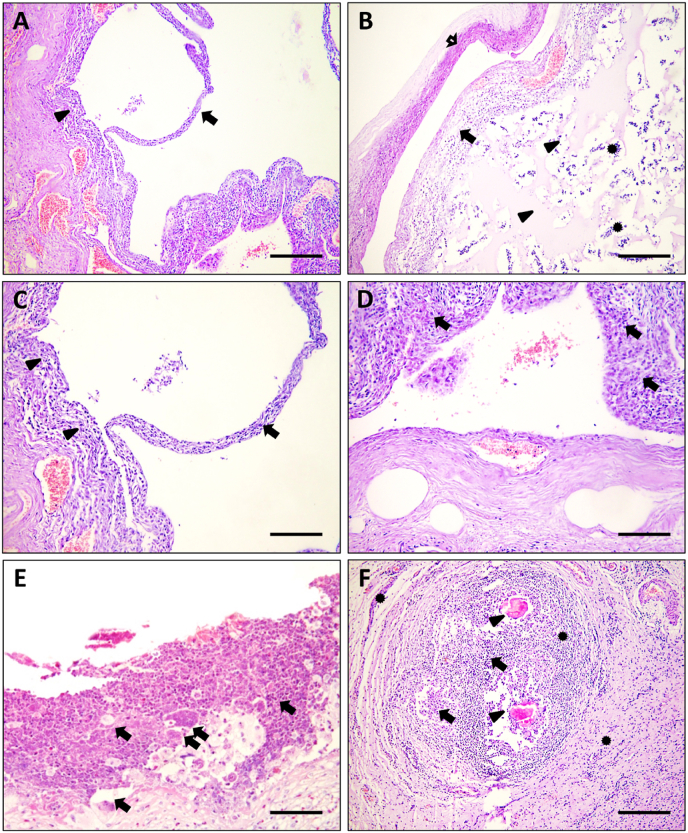
Histopathological sections of cerebrum of Apennine chamois (*Rupicapra pyrenaica ornata*, Neumann, 1899) affected with *Coenurus cerebralis* cyst showing, (A): areolar cyst wall (arrow) with lympho-histiocytic cell infiltrations (arrow-head), scale bar = 500 μm. (B) Cyst membrane (arrow) and liquid, sero-proteinaceous material content of the cyst (arrow-head) with some parasitic debris (asterisks). Note the thinning of the meninges and the inflammatory valley characterized by an abundant infiltration of lymph monocytes, around the cyst (open-arrow), scale bar = 500 μm. (C) Appearance of the lymphohistocytic reaction of the host (arrow) and the cyst wall (arrow-head) at higher magnification, scale bar = 250 μm. (D) Characterization of the peri-cystic inflammatory reaction of the host: many mononuclear infiltrated cells (arrows) are observed, with occasional eosinophil granulocytes; note the presence of some foreign body type multinucleated giant cells (E) (arrows) in the inflammatory host's reaction. Interestingly, in this animal there are no typical “palisading” macrophage organized all around the cystic part, described in sheep and goats, but occasionally very voluminous giant cells, near the cystic membrane and scattered in the lympho-macrophagic infiltrate, scale bar = 250 μm. (F) Some areas of cerebrum of Apennine chamois affected with coenurosis, showed infarction with prominent necrosis and formation of granulomas with material scattered inside (arrows), representing probable aborted parasitic lesions, showing central necrotic-amorphous material of parasitic origin (arrow-head). Note the inflammatory reaction characterized by mononuclear cell infiltrations, in and around necrotic areas, characteristic of type IV hypersensitivity reactions (asterisks), scale bar = 350 μm.

Molecular assay performed on the paraffin-embedded cerebral cyst allowed to detect an amplicon referable to *Taenia* spp. According to Sanger sequencing it was identified as *T*. *multiceps*, showing 99% query cover and 100.00% identity with GenBank accession number LC111535. The obtained sequence has been deposited in GenBank database under the accession number PX241221.

Coenurosis was confirmed as the cause of death based on negative bacteriological cultures, clinical presentation, and specific histopathological findings - including typical inflammatory patterns and neuronal damage - further supported by positive PCR results.

## 4 Discussion

The first documented case of neurocoenurosis in an Apennine chamois (*Rupicapra pyrenaica ornata*), an endangered Italian endemic species, from Monti Sibillini National Park (Central Italy) is described from clinico-pathological and parasitological perspectives.

Based on the gross and histopathological findings in the Apennine chamois, coenurosis was diagnosed, further confirmed by molecular approach applied on the cerebral cyst that allowed to identify *Taenia multiceps*. In fact, except for previous parasitic lesions observed in the liver attributable to inveterate lesions, the only significant pathological conditions correlating with death were found in the CNS. Differential diagnoses for neurological diseases in small ruminants are varied and include non-infectious pathologies, linked to nutritional/deficiency causes such as thiamine deficiency (Cantile and Youssef, 2015), copper poisoning encephalopathy (Sargison et al., 1994; Giadinis et al., 2009) or toxin ingestion (Cantile and Youssef, 2015). Infectious causes include bacterial infections such as listeriosis (*Listeria monocytogenes*), bacterial meningoencephalitis, enterotoxemia and *Ehrlichia ruminantium* (Bedelian et al., 2007); viral agents such as the retrovirus maedi-visna, pseudo-rabies, and rabies (Cantile and Youssef, 2015).

Among parasitic infections, in addition to echinococcosis (Gao et al., 2021; Najjari et al., 2025) and atypical cysticercosis (neurocysticercosis) sustained by *T*. *hydatigena* and *T*. *ovis* (Scala et al., 2016), cerebral coenurosis (*T*. *multiceps*) should be primarily mentioned (Lavin et al., 1995; Moinet et al., 2010).

From a clinical-pathological standpoint, coenurosis is a disease characterized by two phases: an acute phase related to the migration of oncospheres and the development of cysts, which leads to the formation of migration lesions, meningitis, and a mixed inflammatory response, predominantly pyogranulomatous, which is exacerbated by parasite antigens; subsequently, a chronic phase in which, despite persistent inflammatory damage, the mass effect of the expanding coenurus predominates, resulting in pressure-induced ischemic atrophy of the surrounding nervous tissue and a localized fibrous reaction (Giadinis et al., 2012).

This first report of neurocoenurosis in an Apennine chamois highlights the role of wildlife in the epidemiology of *T. multiceps*. In fact, interactions between domestic and wild hosts sharing environmental overlap, particularly in hyperendemic areas, may facilitate the transmission and persistence of the parasite (Bengis et al., 2002). In the case of this species, the restricted range, combined with the high degree of inbreeding, has evident negative repercussions on immunological resistance to infection and adapting to environmental changes, exacerbating the negative impact of several etiological agents (Pedersen et al., 2007). The low genetic diversity and small population sizes in endangered species represent a substantial risk under condition of high parasitic pressure (Pedersen et al., 2007). Specifically, the role of the immune response may be crucial in inducing a widespread lesion, rather than a truly protective response, and may represent the cause of death in the case described. Histopathological lesions have shown the involvement of both cellular (presence of eosinophils, giant cells) and humoral (detection of plasma cells and Splendore-Hoeppli-type bodies) mechanisms. The nervous signs of coenurosis and the, sometimes, lethal effect of the parasite are believed to be the result of a widespread inflammatory process, resulting from toxic and allergic reactions involving type I, II, and IV hypersensitivity phenomena (Edwards and Herbert, 1982). Obstruction of vessels by parasitic elements, inflammation, and parasitic toxins have also been implicated in cerebral necrosis (Mohi El-Din, 2010). Inflammation and necrosis, in the absence of a cyst wall, should be attributed to the migratory phase of the parasitic larvae (Summers et al., 1995). In the present study, macro- and microscopically detectable sterile abscess-like lesions were observed around the cystic area, as also described in other reports, and in other species (Dyson and Linklater, 1979). It has recently been shown that during cerebral coenurosis, profound alterations of the extracellular matrix (ECM) are observed in the brain, characterized by increased expression of type-I, and IV collagen, and in particular aquaporin IV; the increased expression of the latter molecule suggests an important role in the regulation of cerebral edema and the inflammatory response (Karatas and Akcakavak, 2025).

This case appears interesting not only for the anatomo-pathological findings described, but also because it prompts some reflections on the particularly pathogenic and the potential fatal role of this metacestodosis in this new intermediate host, putting at risk conservation concerns in this endangered susceptible species. The Apennine chamois may serve as a novel intermediate host within a sylvatic infection cycle involving the red fox (*Vulpes vulpes*) or the wolf (*Canis lupus*) as definitive hosts (Abbas et al., 2022). However, recent studies on wild carnivores in the Marche region do not detect *T. multiceps* in either species (Crotti et al., 2023a, 2023b). Conversely, at the farm level, Morandi et al. (2020) report that 31% of the investigated farms have sheepdogs testing positive for *T. multiceps* and this ratio directly depend on the number of dogs present on the farm.

Beyond the conservation implications for this endangered species, this emerging scenario carries potential repercussions for livestock economics and human health. As a zoonotic agent, *T. multiceps* can cause cerebral, ocular, and spinal cord coenurosis in humans (Varcasia et al., 2022). Consequently, prevention efforts should focus on regular anthelmintic treatment of shepherd dogs. Implementing such measures every 60 days would effectively disrupt the parasite's life cycle before it reaches the livestock-wildlife interface.

## 5 Conclusions

This study provides the first documented description of *Taenia multiceps* infection in the Apennine chamois (*Rupicapra pyrenaica ornata*), expanding the known host range of this parasite and identifying new vulnerability in protected subspecies. High-pressure parasitic infections are not merely biological curiosities but represent a substantial threat to the conservation of endangered species, where the loss of even a few individuals can threaten population stability. The spatial and environmental overlap between traditional livestock farming and wildlife habitats acts as a critical interface that facilitates the spillover of parasitic infections. In this context, the transmission of *T. multiceps* from domestic dogs to wild ruminants could be facilitated by leaving sheep carcasses in the environment following on-farm slaughter. The systematic performance of necropsies on wildlife is essential and a fundamental pillar for the local and global understanding of the epidemiology of generalist parasites. Without consistent wildlife monitoring, the silent circulation of these pathogens remains undetected, undermining “One Health” efforts to manage disease at the human-animal-environment interface.

## Declaration of generative AI and AI-assisted technologies in the manuscript preparation process

During the preparation of this work the authors used Gemini (Google) in order to improve grammar, language clarity, and sentence rephrasing. After using this tool, the authors reviewed and edited the content as needed and take full responsibility for the content of the published article.

## CRediT authorship contribution statement

**Benedetto Morandi:** Conceptualization, Methodology, Supervision, Visualization, Writing – original draft, Writing – review & editing. **Stefano Gavaudan:** Writing – original draft, Writing – review & editing. **Silvia Crotti:** Formal analysis, Writing – original draft, Writing – review & editing. **Deborah Cruciani:** Formal analysis, Writing – original draft, Writing – review & editing. **Giacomo Rossi:** Formal analysis, Writing – original draft, Writing – review & editing. **Alessio Angorini:** Formal analysis, Writing – original draft, Writing – review & editing. **Fabrizio Dini:** Formal analysis, Writing – original draft, Writing – review & editing. **Alessandro Rossetti:** Writing – original draft, Writing – review & editing. **Federico Morandi:** Supervision, Writing – original draft, Writing – review & editing.

## Declaration of competing interest

All authors declare that they have no competing interests.

## Appendix A Supplementary data

Multimedia component 1

Video S1 Clinical manifestation of locomotor ataxia, traditionally referred to as “staggers,” in an Apennine chamois (*Rupicapra pyrenaica ornata*) secondary to cerebral coenurosis (*Taenia multiceps*). The footage illustrates severe proprioceptive deficits and the classic “staggers” gait, resulting from a space-occupying parasite cyst located within the posterior fossa. The mechanical expansion of the ventricular system induces marked dorsoventral compression of the cerebellum, disrupting central vestibular and proprioceptive pathways. This neuro-anatomical lesion clinically translates into the observed hypermetria, high-stepping gait, and generalized incoordination.
