# Supplementary material for: Metacestodosis as a threat for endangered species: a fatal case of neurocoenurosis in an Apennine chamois (Rupicapra pyrenaica ornata Neumann, 1899) housed in an ex-situ conservation facility within the Monti Sibillini National Park (Italy)
Source: Int J Parasitol Parasites Wildl. 2026 Jun 16;30:101251. doi: 10.1016/j.ijppaw.2026.101251 (PMC13331768; doi:10.1016/j.ijppaw.2026.101251)

**Supplemental material (MRI images of intracranial lesions)**

**Fig. S1.** A 1-centimeter-by-1-centimeter hypointense cystic lesion (yellow arrow) along the course of the third and fourth ventricles.

**
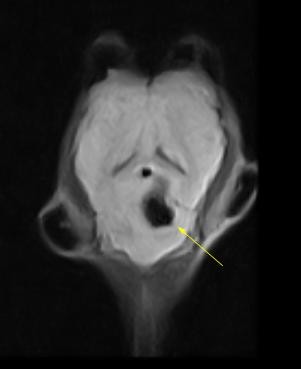
**

**Fig. S2.** Lateral view shows dilated ventricles and compressing the cerebellum in the dorsoventral direction.


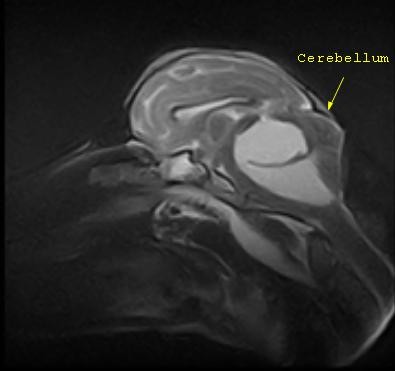

Supplement: Multimedia component 1 [file mmc1.docx]
